# An Update on the Role of Leptin in the Immuno-Metabolism of Cartilage

**DOI:** 10.3390/ijms22052411

**Published:** 2021-02-27

**Authors:** Alfonso Cordero-Barreal, María González-Rodríguez, Clara Ruiz-Fernández, Djedjiga Ait Eldjoudi, Yousof Ramadan Farrag AbdElHafez, Francisca Lago, Javier Conde, Rodolfo Gómez, Miguel Angel González-Gay, Ali Mobasheri, Jesus Pino, Oreste Gualillo

**Affiliations:** 1 SERGAS (Servizo Galego de Saude) and IDIS (Instituto de Investigación Sanitaria de Santiago), The NEIRID Group (Neuroendocrine Interactions in Rheumatology and Inflammatory Diseases), Santiago University Clinical Hospital, Building C, Travesía da Choupana S/N, 15706 Santiago de Compostela, Spain; sitoalcorba@gmail.com (A.C.-B.); maria.gonzalez3112@gmail.com (M.G.-R.); clararf94@gmail.com (C.R.-F.); djidji.aiteldjoudi@gmail.com (D.A.E.); yousof.farrag@gmail.com (Y.R.F.A.); 2 Molecular and Cellular Cardiology Group, SERGAS (Servizo Galego de Saude) and IDIS (Instituto de Investigación Sanitaria de Santiago), Research Laboratory 7, Santiago University Clinical Hospital, 15706 Santiago de Compostela, Spain; francisca.lago.paz@sergas.es (F.L.); javier.conde.aranda@sergas.es (J.C.); 3 SERGAS (Servizo Galego de Saude) and IDIS (Instituto de Investigación Sanitaria de Santiago), The Muscle-Skeletal Pathology Group, Santiago University Clinical Hospital, Building C, Travesía da Choupana S/N, 15706 Santiago de Compostela, Spain; rodolfo.gomez.bahamonde@sergas.es; 4 Epidemiology, Genetics and Atherosclerosis Research Group on Systemic Inflammatory Diseases, Universidad de Cantabria and IDIVAL, Hospital Universitario Marqués de Valdecilla, Av. Valdecilla, 39008 Santander, Spain; miguelaggay@hotmail.com; 5 Research Unit of Medical Imaging, Physics and Technology, Faculty of Medicine, University of Oulu, FIN-90230 Oulu, Finland; ali.mobasheri@oulu.fi; 6 Department of Regenerative Medicine, State Research Institute, Centre for Innovative Medicine, LT-08406 Vilnius, Lithuania; 7 Departments of Orthopedics, Rheumatology and Clinical Immunology, University Medical Center Utrecht, 3584 CX Utrecht, The Netherlands; 8 Department of Joint Surgery, the First Affiliated Hospital, Sun Yat-sen University, Guangzhou 510080, China

**Keywords:** leptin, articular cartilage, chondrocyte, LEPR (ObR), osteoarthritis (OA), rheumatoid arthritis (RA)

## Abstract

Since its discovery in 1994, leptin has been considered as an adipokine with pleiotropic effects. In this review, we summarize the actual information about the impact of this hormone on cartilage metabolism and pathology. Leptin signalling depends on the interaction with leptin receptor LEPR, being the long isoform of the receptor (LEPRb) the one with more efficient intracellular signalling. Chondrocytes express the long isoform of the leptin receptor and in these cells, leptin signalling, alone or in combination with other molecules, induces the expression of pro-inflammatory molecules and cartilage degenerative enzymes. Leptin has been shown to increase the proliferation and activation of immune cells, increasing the severity of immune degenerative cartilage diseases. Leptin expression in serum and synovial fluid are related to degenerative diseases such as osteoarthritis (OA), rheumatoid arthritis (RA) and systemic lupus erythematosus (SLE). Inhibition of leptin signalling showed to have protective effects in these diseases showing the key role of leptin in cartilage degeneration.

## 1. Introduction

Articular cartilage is a highly specialized connective tissue that is found in synovial joints. It consists of a lubricated thin layer of connective tissue between the articulating bones, allowing almost friction-free movement of the joint. It absorbs the compressive and shear forces caused by impact and joint loading and protects the subchondral bone from compression-induced damage [1,2,3].

Cartilage is composed of two parts: extracellular matrix (ECM) and highly differentiated and specialized cells called chondrocytes. The ECM is produced by the chondrocytes, which are the resident cells in the cartilage and represent less than 5% of the total cartilage volume [4]. The ECM is more than 95% of the tissue and consists of water (80% of wet weight), and ECM components include collagens, proteoglycans, non-collagenous proteins, and glycoproteins. Collagen accounts for 60% of the dry weight of cartilage, being type II collagen the 90% of all the collagen in the cartilage. Other collagen types such as type I, IV, V, VI, IX, and XI are present in lower quantities [5]. These tissue-specific cells are derived from mesenchymal stem cells (MSCs) and are specialized in the development, maintenance, and repair of ECM. During the process of growth and development chondrocytes become encapsulated in the ECM that they produce around themselves and are responsible for the maintenance and homeostasis of this matrix., Chondrocytes in mature articular cartilage are terminally differentiated and have a poor ability for replication and ECM repair. The micro-environment of articular cartilage is avascular, aneural and alymphatic, creating a challenge for tissue turnover and ECM repair, which contributes to the slow recovery of cartilage tissue following joint injuries [2,6].

Joint diseases that involve cartilage degeneration are quite common in adults. Osteoarthritis (OA) is the most usual form of arthritis. Many determinants such as trauma, inflammation, age, gender, genetics, and alterations in metabolism are relevant risk factors for OA. However, one of the main factors that increase the risk of OA is obesity. In OA cartilage undergoes various structural changes because of ECM degradation and loss of aggrecan and collagen, which increase the friction of the bones [7]. Chondrocytes become dysfunctional and exhibit abnormal behaviour in many joint diseases, increasing the degradation of the ECM and losing the ability to repair the tissue [8]. In recent years, diverse studies have shown that hypercholesterolemia, hypertension, and high circulating levels of glucose and insulin play a key role in joint and cartilage homeostasis. Despite this evidence, the mechanisms behind the catabolic and inflammatory process are extraordinarily complex and still poorly defined. Recent studies suggest that leptin, which is a hormone prevalently synthesized by white adipose tissue (WAT) depending on the levels of fatty acids in the blood, has an especially prominent role in joint disease pathogenesis [9].

In this review, we will focus on the role of leptin in the regulation of cartilage homeostasis and its implication in the development of degenerative joint diseases such as OA.

## 2. Physiology and Pathophysiology of Leptin

Leptin is a hormone prevalently produced by adipocytes in WAT, considered as a regulator adipokine of various physiological and pathological processes. This hormone is a 16kDa non-glycosylated protein encoded by the gene *LEP* (human *Ob* gen homolog) [10]. Leptin levels in the blood are proportional to adipose tissue mass. Its physiological effects occur when this hormone appears at low levels. Leptin deficiency or resistance in human patients causes severe obesity, diabetes, and infertility with hypogonadism [11].

Leptin synthesis and secretion are regulated by multiple factors, including the intake of food and the action of various hormones such as insulin, steroid hormones, and norepinephrine. Glucocorticoids act directly on WAT and have the greatest stimulatory effect on leptin secretion [12]. Inflammation cytokines also play a significant role in leptin secretion since the activation of the COX-2 pathway due to increased Prostaglandin E_2_ (PGE_2_) and arachidonic acid (AA) induce leptin synthesis. Insulin induces leptin secretion during food intake in a hyperglycaemia-independent way [13]. Adenylate cyclase activators and melatonin have been reported to lead leptin production to decrease [14].

Since the discovery of leptin in 1994 by Friedman’s group, this class I adipocytokine has been considered as a bodyweight regulator hormone. It can reduce food intake and increases energy expenditure, by inducing anorectic factors such as CART or POMC and inhibiting orexigenic factors such as NPY or AgPR. The discovery of its involvement in multiple biological processes like angiogenesis, haematopoiesis, regulation of bone mineral density, and homeostasis of the lymphatic organs and the immune system turned its physiological role into a pleiotropic factor [15,16]. 

Interestingly, recent studies have shown that leptin, along with other adipocytokines such as adiponectin, resistin or visfatin, are potent modulators of immune responses [17]. Leptin levels are related to autoimmune disorders such as multiple sclerosis, rheumatoid arthritis, systemic lupus erythematosus or inflammatory bowel diseases. Low levels of leptin expression due to congenital deficiency or starvation provokes immune dysfunction, which is reversible with leptin administration. Its overexpression occurring in obesity leads to increased susceptibility to autoimmunity and chronic inflammation, evidencing the role of leptin as a pro-inflammatory hormone [18,19]. Leptin is a soluble molecule whose function is mediated by binding to a specific receptor encoded by the *LEPR* gene (*ObR* human homologous gene). This gene codes for six different isoforms of the receptor due to alternative splicing, producing proteins with differing forms and molecular weights. The short forms are expressed in various parts of the body such as gonads, liver, lung, or vascular endothelium. It turns out that these isoforms are not able to transduce the signal of the hormone, and only the long isoform (LEPRb) is able to signal properly [20]. LEPRb is expressed in the hypothalamus, where its main function is to regulate body weight. Furthermore, the long isoform is expressed in other tissues such as pancreatic beta cells, foetal liver ovarian follicular cells, T lymphocytes, osteoblast, vascular endothelial cells and CD34+ hematopoietic bone marrow precursor [10,21].

**Figure 1:**
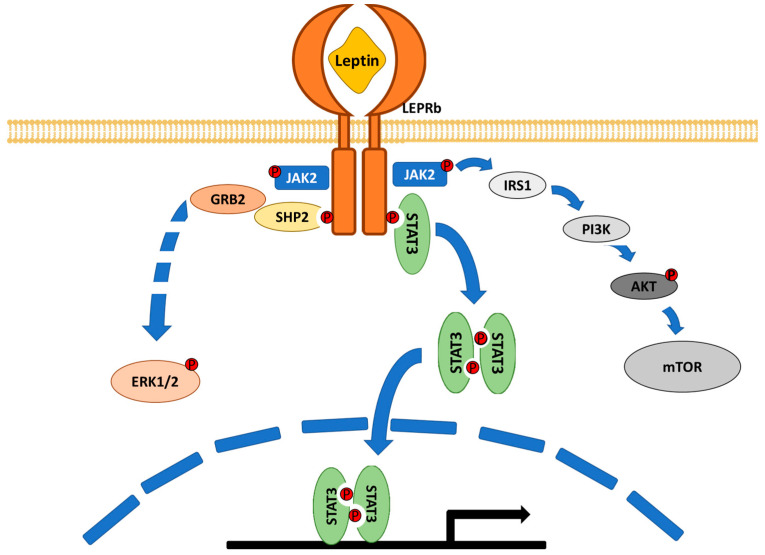
Schematic representation of leptin signalling. There are different isoforms of the leptin receptor, but only the long receptor (LEPRb) has shown to transduce completely the leptin signalling. Leptin induces LEPRb dimerization and phosphorylation. This receptor activation produces the activation of the JAK2/STAT3 pathway but also the activation of MAPK and PI3K pathways.

The interaction between leptin and the receptor (LEPRb) induces conformational changes in the receptor that promote the activation of different signalling pathways, mainly the JAK2/STAT3 pathway, but other pathways are at play such as the MAPK pathway through the activation of ERK1/2 and the PI3K pathway through IRS1 phosphorylation [22,23] (Figure 1).

### 2.1. Leptin in Pathologies of Articular Cartilage

Leptin is a soluble molecule that can diffuse into the synovial fluid from the blood, but in cartilage, leptin is also produced by chondrocytes themselves but also by periarticular tissues such as infrapatellar fat pad and synovia [24,25]. Interestingly, infrapatellar fat pad (IFP), produce adipokines, such as leptin, chemerin and visfatin [26,27]. To note, the elevated expression of these factors in OA IPFP and synovium highlights the potential role of adipokines in the initiation and perpetuation of synovitis and OA symptoms [28,29]. This aspect further confirms that adipokines, coming from intra-articular tissues, may also affect cartilage homeostasis, as widely proven by in vitro evidence previously published by our group and others.

Leptin levels in cartilage are higher in OA and RA than in healthy joints [30,31]. Generally, the influence of obesity on cartilage degradation has been related to abnormal biomechanical loading. However, it has also been reported that obesity is related to the development of OA in non-weight bearing joints [32,33]. Therefore, it is reasonable to propose that obesity-associated protein factors such as leptin, as well as other adipokines, are likely involved in the development of articular degenerative inflammatory diseases. [34]

### 2.2. Leptin in Chondrocyte Proliferation and Metabolism

Human chondrocytes express the LEPRb receptor, suggesting that leptin may have significant effects on these cells. In addition, experiments with exogenous leptin administration in rat knee joints induce the phosphorylation of STAT1 and STAT5 in chondrocytes and increase the proliferation and secretion of proteoglycans. Although these experiments initially suggested that high circulating leptin levels may protect cartilage degeneration in short-term periods [35,36,37], the response of human chondrocytes to continuous exposures of leptin, as occurs in obesity, showed a reduction in cell viability. This also occurs in chondrocytes derived from OA cartilage [24,38]. The overexpression of LEPRb in human chondrocytes, in the presence of leptin, increases senescence through the activation of mTOR [39]. These reports suggest that prolonged activation of the leptin pathway in chondrocytes reduces cell viability and might induce cartilage degeneration, while a low and short activation could have protective effects.

Recent experiments using rat chondrocytes showed that leptin can induce chondrocyte apoptosis by stimulating LOXL3 expression, activating the mTOR pathway and inhibiting chondrocyte autophagy [40]. The JAK2/STAT3 signalling inhibits chondrocyte apoptosis, suggesting a principal role of this pathway in leptin-induced chondrocyte death [41]. Another pathway that may be involved is the activation of JNK by leptin mediated DUSP9 downregulation. Experiments that overexpress DUSP9 showed to partially inhibit chondrocyte apoptosis induced by leptin [42]. Many proteins such as SOCS-3 and vaspin inhibited leptin signalling in experiments with animal models [43,44].

Leptin controls cartilage homeostasis. This hormone not only affects chondrocytes viability but also controls chondrocytes secretion. Leptin induces the secretion of metalloproteinases (MMPs) by the chondrocytes and specifically induces MMP1, MMP3, MMP13, ADAMTS, and ADAMTS5, which are responsible for the cleavage of collagens, elastin, and aggrecans [45]. 

### 2.3. Leptin in Cartilage Inflammation

Leptin can induce the release of pro-inflammatory agents in cartilage. Experiments carried out with OA chondrocytes show that leptin can influence the synthesis of NO through the induction of inducible NOS [46]. The increase of NO in the cartilage induces the expression and activation of MMPs [47], inhibits the synthesis of proteoglycans and collagen [48,49], induces the death of chondrocytes [50] and the inflammatory response by increasing the levels of COX2, PGE_2_ and pro-inflammatory cytokines such as IL-6 and IL-8 and decreasing levels of TGF-β, IL-1β receptor antagonist (IL-1ra) and IL-10 [51].

Studies conducted with bovine and human cartilage have shown that the effect of leptin on the degradation of the collagen of the ECM is increased when combined with pro-inflammatory cytokines such as IL-1β or TNF-α, due to increased secretion of MMPs by chondrocytes. This is because leptin induces the phosphorylation of tyrosine residues of STAT1, 3 and 5, but not the serine residues, whereas other pro-inflammatory cytokines can induce the phosphorylation of these residues, increasing the global response [29].

Leptin pathway signalling has been described as a powerful regulator of the immune system, with most immune cells expressing the LEPR receptor being leptin dysfunction associated with impaired cell-mediated immunity [52]. Lack of leptin in mice (*ob/ob*) or acute starvation develop thymus atrophy, T cell lymphopenia and spleen reduction, which are reversible with exogenous leptin administration, proving that leptin signalling is necessary for correct immune organ function [53].

**Figure 2:**
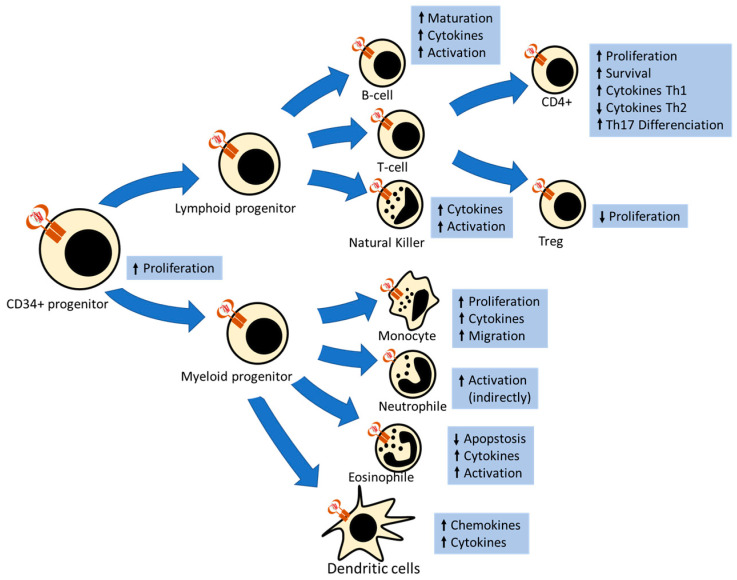
Schematic representation of the leptin effect in hematopoietic derived cells. Hematopoietic CD34+ progenitor and all derived cells express the long leptin receptor (LEPRb), except neutrophiles, which only express the short receptor (LEPRa). Leptin signalling induces proliferation, activation, cytokine secretion and migration in myeloid-derived cells. Leptin induces maturation, cytokine secretion and activation of lymphocytes B. In lymphocytes T, leptin induces survival and proliferation of naïve CD4+ cells and Th1 and Th17 response and repress Th2 response and Treg proliferation.

Hematopoietic CD34+ progenitor cells—that can differentiate into myeloid and lymphoid derived cells—express long and short forms of the leptin receptor. Experiments with leptin-deficient mice suggest that leptin induces the proliferation of these cells [54]. Interestingly, this proliferation signal is conserved in CD34+ derived cells [52] (Figure 2).

Studies with human peripheral blood mononuclear cells (PBMCs) showed that leptin can induce monocyte proliferation, by inducing GM-CSF expression; increasing monocyte activation markers such as CD38, CD25 or CD71 and LPS response; and stimulating cytokines secretion, such as IL-6 and TNF-α [55]. AlsoFurthermore, leptin can induce lipid bodies released in these cells [56]. Later studies showed that leptin has chemoattractant properties for monocyte and macrophages that are mediated by LEPR long isoform activation and PI3K signalling. This discovery suggested that there are leptin gradients that can guide macrophage tissue extravasation [57], but assays with LEPR KO mice showed that leptin is not the only cytokine involved in this process [58].

Analysis with peripheral blood eosinophilic cells showed that these cells express long and short forms of LEPR. Leptin signalling delays eosinophil apoptosis in a GM-CSF independent way by inducing PI3K and p38 MAPK signalling pathways [59]. Leptin induces eosinophil activation, increasing the synthesis of lipid bodies and eicosanoids, such as LTC4, mediated by mTOR activation. Mast cells express LEPR and leptin signalling promotes the release of inflammatory mediators or cytokines, such as PGD2 and TNF-α, which are necessary for leptin-induced eosinophilic activation and migration [60].

Polymorphonuclear neutrophils (PMNs) only express the LEPR short receptor (LEPRa). This receptor cannot transduce leptin complete signalling, but some studies suggested that it may induce the MAPK pathway [61]. When these cells are stimulated with leptin, the ROS production increases, suggesting an activation role of leptin in these cells [62]. Leptin cannot activate neutrophils directly. Leptin-driven activation increases and promotes TNF-α and IL-6 secretion by monocytes [63]. Despite of this, recent studies suggest that physiological leptin concentrations are not enough to induce leptin effects in neutrophils showing the indirect effect of this hormone [64].

Regarding NK cells, short-term leptin induction revealed an increment of cytokines secretion such as interferon-gamma (IFN-γ), and activation markers such as TRAIL or CD69. However, long-term leptin induction inhibits these effects and reduces cell proliferation [65,66,67]. Notwithstanding, experiments carried out with depleted leptin mouse models showed that this hormone is necessary for NK cell function [68].

Dendritic cells (DC) are the most important antigen-presenting cells. These cells express the LEPRb receptor, inducing the production of cytokines and chemokines, such as IL-1β, IL-6, IL-12 and TNF-α, by leptin signalling. However, leptin does not change the phenotypical maturation of DCs or LPS-induced maturation. Leptin has an antiapoptotic effector in DC by enhancing NF-κB activation. Leptin induces MIP-1α expression that produces an M1 and Th1 activation response [69].

T lymphocytes express LEPRb receptor [70]. T cells from starving mice had defective pro-inflammatory cytokines secretion and glucose uptake due to leptin privation. These results prove that leptin is necessary for correct T cell function [71]. In starvation, CD4+/CD8+ and CD4+/CD8- cells are reduced. Leptin administration showed an increase in CD4+ T cell number, inducing the differentiation of CD4+/CD8+ cells into T helper cells (CD4+/CD8-) and promoting CD4+ survival and proliferation. This is because CD4+ cells have more LEPRb expression than CD8+ cells [72]. In these cells, leptin showed a clear induction in cytokine pro-inflammatory TH1 secretion, such as IFN-γ, and a reduction in anti-inflammatory TH2 phenotype, such as IL-4 [73]. Leptin has shown to decrease the survival of Treg (CD4+ CD25+), which suppresses the proliferation of CD4+CD25− T cells [74]. Th17 cells have a significant role in the maintenance of inflammation and autoimmunity. Studies performed with leptin knock-out mice (*ob/ob*) showed that leptin is necessary for the STAT3 dependent Th17 differentiation from CD4+ naïve cells [55].

B cells express LEPRb on the cell surface, leptin signalling in leptin deficient mice (*ob/ob*) showed that these animals are protected against lupus development after pristane injection. These findings suggest a key role in disease, caused by immune overresponse, manifestation [75]. Recent studies revealed that leptin reduces antibody secretion, due to mTOR activation, and reduced class switch in B-cells [76]. Leptin represses apoptosis in these cells by inducing BCL-2 and cyclin D1 expression, and induces secretion of pro-inflammatory cytokines, such as TNF-α and IL-6, and reduces anti-inflammatory secretion IL-10 [77,78]. 

## 3. The Role of Leptin in Pathological Joint Diseases

### 3.1. Leptin in Osteoarthritis

OA is the most frequent arthritis form, affecting 500 million people globally [79]. This disease is characterized by structural alterations in the cartilage, subchondral bone, ligaments, capsule, synovium, and periarticular muscles. In OA, there is an imbalance between the destruction and repair of cartilage, which finally provokes cartilage loss and bone erosion. This disease is associated with many risk factors such as age, sex, trauma, obesity, and genetics that may have a significant role in disease development and progression [80].

The relationship between leptin levels in serum and OA is controversial. Many studies reported that elevated levels of serum leptin are related to OA, especially in advanced disease patients [81,82,83]. On the contrary, other studies argue that there is no relationship between leptin circulating levels and OA progression [84,85]. Leptin levels are related to body mass index (BMI). Interestingly, body weight has an influential role in knee OA development not only for the mechanical stress in the joints, [86,87] but also because there is an increase of pro-inflammatory adipokines and cytokines by WAT.

**Figure 3:**
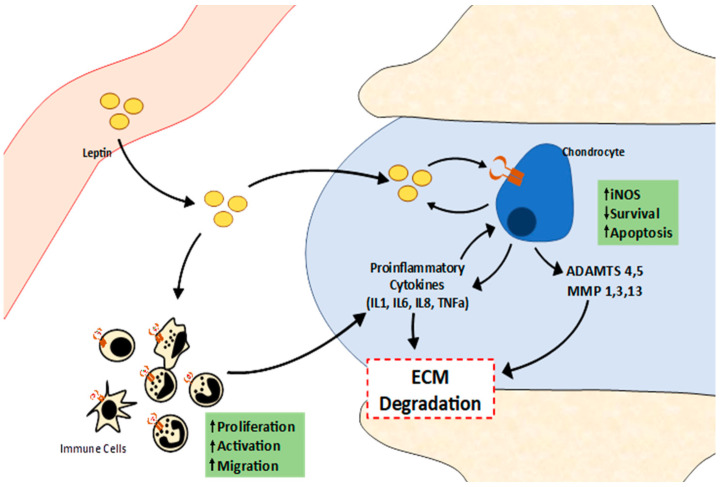
Schematic representation of leptin signalling in cartilage. The leptin from blood can get into the synovial cavity, where it decreases chondrocyte survival and induces apoptosis, and elevates NO production, and IL6 and IL8 secretion by chondrocytes. This activity combined with metalloproteinases (MMP) secretion by chondrocytes induces extracellular matrix (ECM) degradation. Leptin also can activate immune cells and induce its proliferation and migration to articulation, inducing pro-inflammatory activity in the tissue that contributes to ECM degradation.

Analysis of leptin levels in synovial fluid shows that elevated levels of leptin are related to BMI and OA progression (Table 1) [81,88,89,90]. Leptin levels in synovial fluid may be caused by the diffusion of adipokine from blood into the synovial tissue, but studies with human chondrocytes showed that these cells can express leptin and its long receptor, suggesting a local role of this adipokine [27] (Figure 3). Despite of this, recent studies using mouse lipodystrophy models show that these mice are protected against OA caused by overweight or cartilage damage, but when these mice recover the adipose tissue, recover the susceptibility to OA [91]. This evidence shows that adipose tissue is necessary for OA development, but more experiments are necessary to find the role of this tissue in OA progression.

Other studies that evaluate knee OA risk in leptin knockout mouse models, showed that, in the absence of leptin, weight has any influence on knee OA prevalence [92]. 

Leptin expression by chondrocytes is low in normal or low damaged cartilage, but these levels increased with OA progression, being higher when the cartilage is more severely damaged. This leptin increase is followed by an increase of LEPRb receptor expression, suggesting that leptin signalling is higher when more advanced is the disease [24].

Leptin exogenous administration in OA derived chondrocytes decreased cell viability by inducing chondrocyte apoptosis, which is due in part to the increase of ROS production. To note, inhibition of the main leptin signalling pathway JAK2/STAT3 decreased ROS production and chondrocyte apoptosis [41]. Other studies show that leptin can induce the production of NO, PGE_2_, IL-6, and IL-8 in human osteoarthritic cartilage. Leptin can induce the expression of NOS in chondrocytes due to activation of JAK2/STAT3, JNK, and NF-κB pathways. NO, alone or in combination with IL-1β, can increase PGE_2_, pro-inflammatory cytokines (IL-2 and IL-8), and VCAM-1, inducing cartilage inflammation and immune cells extravasation, which promote cartilage degradation [46,51,93]. Other studies showed that leptin induces, with IL-1, the expression of the ELF3 transcription factor, which induces NOS2, COX2, and MMP13 expression and represses COLA1 transcription, confirming the catabolic role of leptin in OA [94]. In OA, the chondrocytes increase the synthesis and release of ECM degradative enzymes, such as MMP1 and MMP13 [29], and ADAMTS-4, ADAMTS-5, and ADAMTS-9 [95], inducing cartilage degeneration. This secretion is mediated by activating JAK/STAT, MAPK and NF-κB pathways. This suggests a key role of leptin in cartilage degeneration during OA development and disease progression.

### 3.2. Leptin in Rheumatoid Arthritis

Rheumatoid arthritis (RA) is a chronic disease that affects approximately 0.25% of the global population, being more frequent in women than in men [96]. RA is an autoimmune disease that induces cartilage destruction. In this disease, leucocytes and other immune cells infiltrate into the synovial compartment. Synovial fibroblasts develop an inflammatory phenotype and chondrocytes enhance cartilage catabolism, promoting synovial destruction, and cytokines increase in the synovial fluid such as TNF-α, IL-6 and IL-1β. [97].

Blood leptin levels are increased in patients with RA in comparison to healthy controls [98,99] (see also Table 1). Disease activity score value (DAS28) and articular erosion are causally related to leptin serum levels. Interestingly, as occurs in other degenerative articular diseases, synovial leptin levels are related to RA development and disease stage [100,101]. 

Experiments carried out in rodents lacking leptin expression or leptin receptors showed that these animals have less antigen induced arthritis ability, proving that leptin signalling increases synovial inflammation [102]. Leptin injection in synovial joints of RA experimental mice worsened articular degeneration.

In RA, many cells such as lymphocytes, macrophages, and fibroblast-like synoviocytes (FLSs) are activated. Nevertheless, the most important effector in synovial hyperplasia is FLSs cells. These cells can secrete inflammatory mediators such as VEGF and IL-6. Synovial FLS cells are involved in the control of cartilage structure and dynamics in normal tissue, but in RA these cells increase their number and became cartilage-destruction cells [103]. Experiments using human RA FLSs cells showed that leptin does not affect RA FLS activation but can induce cell migration by increase ROS production in synovial tissue, increasing chemotactic cytokines such as TNF-α, IL-6, and IL-1β [104,105].

As previously described, the increase in leptin levels not only has an effect by increasing the catabolism of chondrocytes in cartilage, but also induces the proliferation and activation of cells of the immune system [52]. In particular, leptin increases IL-17A, IL-17F, NLRP3, and IL-1β and increases the number of Th17 cells [106]. Elevation of Th17 cell numbers is an important event in RA disease. These pro-inflammatory cells lead to the induction of inflammation, cellular infiltration and cartilage degradation mediated by IL-17 secretion [107]. AlsoFurthermore, leptin inhibits the differentiation of Treg cells, which are a potent inhibitor of autoimmunity [108]. This suggests that leptin has a prominent role in the immune response during the development, establishment, and progression of RA.

### 3.3. Leptin in Systemic Lupus Erythematosus (SLE)

Systemic lupus erythematosus (SLE) is an autoimmune disease that affects multiple organs with several nervous system involvements. This disease is produced by self-antigen recognition by immune cells [109].

Recent studies show that leptin levels in plasma are increased in patients with SLE compared with controls (see also Table 1) [110,111]. Leptin overexpression decreases Treg in SLE patients. Experiments carried out with Treg from SLE patients show that leptin blockage increases Treg expansion capability [112]. This suggests that leptin inhibition can reduce autoimmunity in SLE patients. 

Experiments using mouse models showed that in lupus-like induced diseases mediated by pristane injection, high leptin levels have an increased response of pristane generated IgG secretion and accelerate lupus in NZB/W mice or MRL/Mp-Faslpr mice, which develop SLE spontaneously. However, leptin deficient mice or those treated with leptin antagonists exhibit no response to pristane injection [75,113]. These results have shown that leptin has an important role in SLE development due to the stimulation of autoimmunity. 

### 3.4. Leptin in Psoriatic Arthritis

Psoriatic arthritis (PsA) is a chronic disease that appears in 33 to 50% of the patients affected with psoriasis [114]. This disease is caused by the overactivation of T cells in the cartilage tissue, that secret Th1 pro-inflammatory cytokines such as IFN-γ, TNF-α and other interleukins [115].

Overweight and obesity lead to a higher risk of developing PsA and increase the severity of the lesions in the cartilage [17]. AlsoAdditionally, leptin serum levels were shown to be related to PsA development and severity [116,117,118]. However, there are no published studies that have evaluated the impact of leptin in PsA cartilage, highlighting the need for further research. 

## 4. Leptin as a Biological Therapy

Some studies have suggested that excess dysfunctional fat mass is a risk factor for OA. Indeed, overweight, and obese adults have significantly increased plasma levels of classic pro-inflammatory cytokines such as TNF-α, IL-1β, and IL-6, but also of leptin and other adipokines. The abnormally-elevated levels of these factors in OA patients are a crucial factor contributing to cartilage loss in these subjects. Although anti cytokines therapy in rheumatoid arthritis is currently one of the best therapeutic modalities, in OA it is still in an early phase since the number of clinical studies evaluating the efficacy of biologics in OA is still too small. Taking into account all these observations, it could be possible to guess whether targeting pro-inflammatory adipokines such as leptin may be effective in cartilage inflammatory degenerative diseases. 

The possibilities to antagonize leptin actions by using high-affinity leptin-binding molecules—by blocking the leptin receptor with monoclonal humanized antibodies or by administering leptin mutants able to bind the leptin receptor without activating it—are probably feasible. It is necessary that these antibodies should not change the activity of leptin on food intake, to avoid the development of hyperphagia and obesity. Currently, there is a recombinant form of leptin, which is developed as a biological drug for human therapy (Metreleptin, Myalept®). This synthetic hormone is formed by the complete leptin protein with an additional methionyl residue at the N-terminal end. This drug has been approved by the FDA for the treatment of lipodystrophy in a congenital or acquired generalized form (non-HIV-related), but not for the partial forms [119]. Despite the regulatory approval of this biological drug, no clinical trials have been performed in joint diseases, so further studies on the effects of this drug on cartilage degeneration are needed.

As previously described, leptin is shown to be an immunostimulatory hormone that induces cartilage destruction under pathological conditions. For these reasons, targeting leptin signalling would be a potential therapy for degenerative diseases of cartilage. Recently, the development of leptin receptor antagonists such as PEG-MLA [120] and mAb-9F8 [121] have suggested that attenuating leptin signalling, and the associated immune response may be an attractive approach for targeting leptin-induced cartilage degradation.

Another way to reduce leptin signalling is by reducing its levels in the blood by decreasing body weight, specifically adiposity. Studies based on losing weight through fasting, nutrition control and pain managed techniques in overweight patients were useful to decrease leptin and IL-6 levels and reduce OA and RA severity [122,123,124]. The available evidence suggests that weight loss combined with physiotherapy may be a good approach for degenerative joint diseases and may delay joint replacement surgery. 

## 5. Conclusions

It is now clear that leptin has multiple relevant roles in the body, and many research efforts are driven to elucidate the intricate network among, leptin metabolic disorders, inflammatory diseases, and the immune system. Although many aspects are still unclear, this review summarizes the current knowledge on the role of leptin in cartilage pathophysiology and in certain rheumatic diseases. 

Leptin has clear catabolic effects in articular cartilage, however certain aspects of its involvement in joint degeneration are still not well understood. 

The data presented here suggest that leptin could be considered a link between immunometabolism and rheumatic diseases and regulation or modulation of its signalling pathways may represent innovative therapeutic strategies for autoimmune and rheumatic disorders. Nevertheless, further insights into the intimate mechanisms regulating peripheral and central leptin activity will be of great relevance for future therapeutic strategies. 

## Figures and Tables

**Table 1:** Summary of all cited studies about the role of leptin in human cartilage diseases.

Author	Year	Disease	Patients	Sample Origin	Leptin Relation with Disease
Simpoulou, T. et al. [24]	2007	OA	11	Serum and synovial fluid from Knee and Hips cartilage	Leptin and leptin receptor levels are increased in OA disease and disease progression
Calvet, J. et al. [81]	2016	OA	115	Synovial fluid from Knee	Leptin levels are increased in OA severity
Kroon, F.P.B et al [82]	2019	OA	6408	Serum	Leptin levels are increased in OA disease
Massengale, M. et al. [84]	2019	OA	2477	Serum	No evidence of relation
Yusuf, E. et al. [85]	2011	OA	164	Serum	No evidence of relation
Xiong, H. et al. [89]	2019	OA	38	Synovial fluid from temporomandibular articulation	Leptin levels are increased in OA disease
Lübbeke, A. et al. [90]	2013	OA	219	Synovial fluid from hips and Knees	Leptin levels are increased in OA and are related with join pain
Min, S. et al. [88]	2021	OA	148	Serum	Leptin levels are increased in OA disease
Rho, Y.H. et al. [90]	2009	RA	167	Serum	Leptin levels are increased in RA disease
Wang, M. et al. [99]	2017	RA	54	Serum	Leptin levels are increased in RA
Olama, S.M. et al. [100]	2010	RA	40	Serum and synovial fluid from Knee	Leptin levels are increased in RA
Afifi, A.E. et al. [110]	2020	SLE	50	Serum	Leptin levels are increased in SLE
Lee, Y.H. et al. [111]	2018	SLE	1333	Serum	Leptin levels are increased in SLE
Wang, X. et al. [112]	2017	SLE	47	Serum	Leptin levels are increased in SLE
Takahashi, H. et al. [116]	2008	PsA	122	Serum	Leptin levels are increased in PsA
Çerman, A.A. et al. [117]	2008	PsA	43	Serum	Leptin levels are increased in PsA and related with disease severity
Xue, Y. et al. [118]	2012	PsA	41	Serum	Leptin levels are increased in PsA

## Abbreviations

ECM	extracellular matrix
WAT	white adipose tissue
OA	osteoarthritis
RA	rheumatoid arthritis
DC	dendritic cells
SF	synovial fluid
SLE	systemic lupus erythematosus
